# From Prevention to Care: Unveiling a Parasitic Leiomyoma Within a Gigantic Abdominal Mass

**DOI:** 10.7759/cureus.47361

**Published:** 2023-10-20

**Authors:** Teresa Pipa, Carla Moreira, Carolina Rodrigues, Daniela Azevedo, Fernando Albuquerque, Filipe Fernandes, Teresa Melanda, Ana Marques, Inês Figueiredo, Rita Nunes

**Affiliations:** 1 Family and Community Medicine, Unidade de Saúde Familiar (USF) Lusitana, Viseu, PRT

**Keywords:** parasitic leiomyomas, leiomyomas, gynecologic cancers, primary healthcare services, intra-abdominal mass, cervical cancer screening

## Abstract

Leiomyomas are non-cancerous tumors emerging from the smooth muscle cells and fibroblasts of the myometrium. They are the most common pelvic tumors in females and are usually asymptomatic. Parasitic leiomyomas have been defined as unusual variants of pedunculated leiomyomas. When symptomatic, leiomyomas can cause abnormal uterine bleeding, pelvic pain/pressure, and reproductive effects, such as infertility or adverse pregnancy outcomes. Treatment varies depending on age, symptoms, and the preference to preserve fertility.

In this article, we describe the case of a 58-year-old woman who presented for a scheduled cervical cancer screening in primary healthcare. Upon objective examination, the patient exhibited a distended and tense abdomen, along with edema in the lower limbs. These symptoms were associated with fatigue and weight gain over the last few months. Subsequent investigation led to an exploratory laparotomy which revealed a massive abdominal mass, measuring approximately 45 cm in diameter and weighing 35 kg. The findings were suggestive of a parasitic leiomyoma.

## Introduction

Leiomyomas are the most common solid pelvic tumors in women [1]. They are mostly asymptomatic and exhibit slow growth. Symptoms are related to the number, size, and location of the tumors and include menorrhagia, infertility, recurrent pregnancy loss, pelvic pain/pressure, polycythemia, impingement, and related complications [2]. Treatment depends on a woman’s age and her desire to maintain fertility. Symptomatic women who do not intend to or can no longer become pregnant should undergo total abdominal hysterectomy and bilateral salpingo-oophorectomy [2].

Cervical cancer screening is an important aspect of women’s healthcare that offers early detection and intervention, ultimately leading to improved outcomes. In primary healthcare in Portugal, every woman aged 25 to 60 is invited for screening, and if both their cytology results and human papillomavirus test are normal, they are subsequently recalled every five years [3]. The following clinical case discusses a patient’s routine screening visit for cervical cancer and how any visit to primary healthcare can and should be used to assess the presence of complaints that warrant timely counseling. The call to primary healthcare for screening facilitated the redirection of an acute, life-threatening, and rare condition, unrecognized by the patient.

## Case presentation

A 58-year-old Caucasian woman, with six years of education, was employed as an administrator in a construction company. She was married and resided in an extended family household, living with her parents, husband, daughter, and grandson. Socioeconomically, she belonged to class III (middle) according to the Graffar scale. Her medical history included an acute myocardial infarction, dyslipidemia (evidenced by xanthelasmas), anxiety, obesity, hypertension, and an umbilical hernia. She denied any smoking or drinking habits. Her current medication regimen consisted of bisoprolol 5 mg + perindopril 5 mg OD, amlodipine 10 mg OD, rosuvastatin 20 mg OD, acetylsalicylic acid 100 mg OD, and alprazolam 1 mg OD. There was no relevant family history. The following gynecological and obstetric history was reported by the patient: menarche at 12 years old; regular menstrual cycles lasting two to three days with normal flow and without dysmenorrhea or dyspareunia; three pregnancies including two live births and one miscarriage; and menopause at 52 years of age without hormonal therapy.

**Figure 1:**
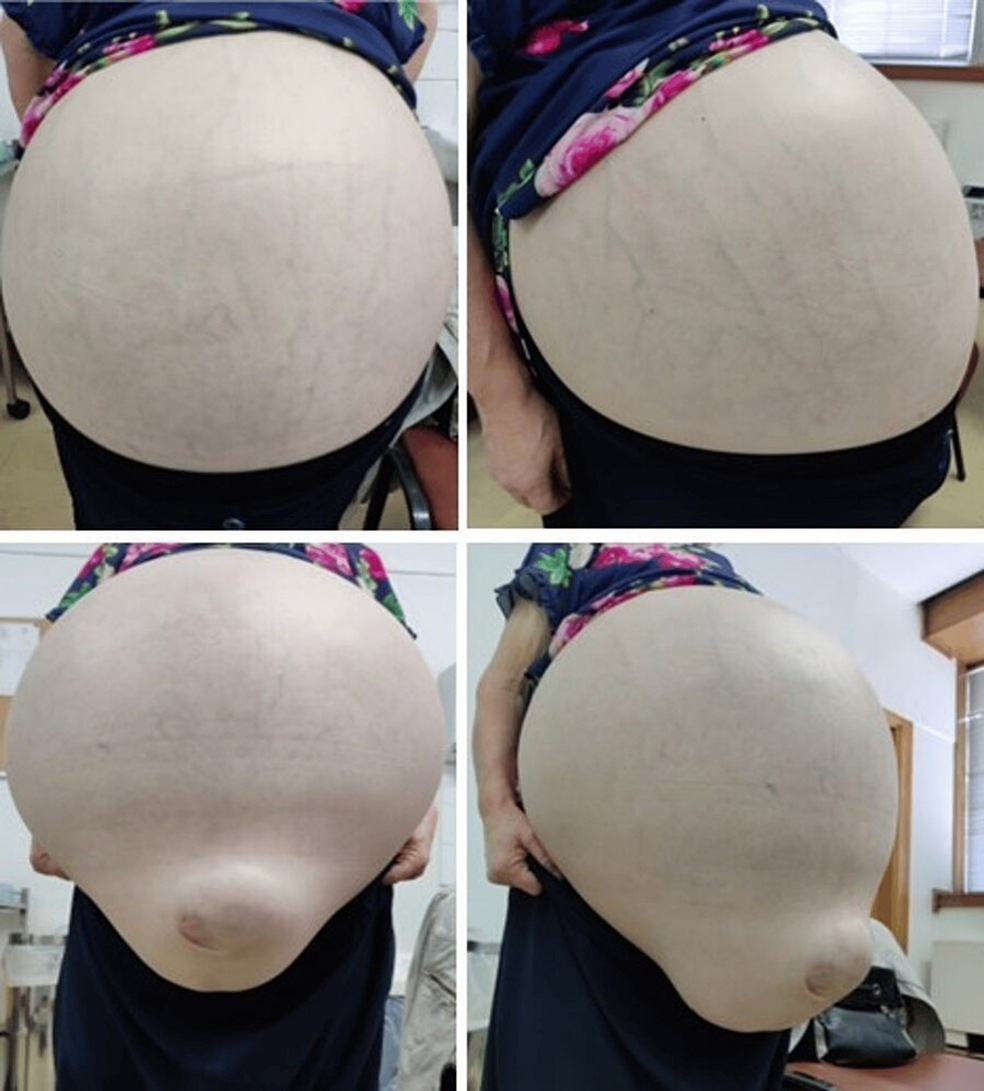
Frontal and lateral photos of the distended abdomen with an umbilical hernia.

On 01/07/2021, the patient was scheduled for a cervical cancer screening. Due to the COVID-19 pandemic, the patient no longer had direct contact with primary healthcare services, except for non-presential consultations to renew medication since February 2020; however, she had never been a highly frequent attendee of primary healthcare services. Upon entering the medical office, it was evident that her abdomen was significantly distended, contrasting with her slim arms and legs. She reported that over the past three months, her abdomen had gradually enlarged and become swollen. She mentioned adhering to a dietary plan supervised online by a nutritionist, and although she perceived a sense of weight loss, she had actually gained 10 kg in three months. Additionally, she complained of fatigue. She denied changes in bowel habits, urinary patterns, postmenopausal bleeding, or abdominal pain. While preparing for the cytology test, the patient experienced tachypnea and dyspnea, leading to the decision to postpone the examination. Upon objective examination, the patient exhibited cutaneous pallor, an oxygen saturation of 98% while breathing ambient air, a blood pressure reading of 145/80 mmHg, a weight of 95 kg, and a body mass index (BMI) of 41 kg/m^2^. While conducting the abdominal examination, a visual inspection revealed a visibly distended abdomen displaying telangiectasis and no indication of thrombocytopenic purpura. The abdominal circumference measured 143 cm, and a noticeable umbilical hernia was also evident (Figure 1).

Palpation of the abdomen revealed tension, poor depressibility, and painlessness, accompanied by a positive fluid wave sign. The lower limbs were cold and showed generalized edema, despite the absence of signs of venous insufficiency. The remainder of the physical examination yielded normal findings. Consequently, the patient was referred to the emergency department of the designated reference hospital for further evaluation and management.

**Figure 2:**
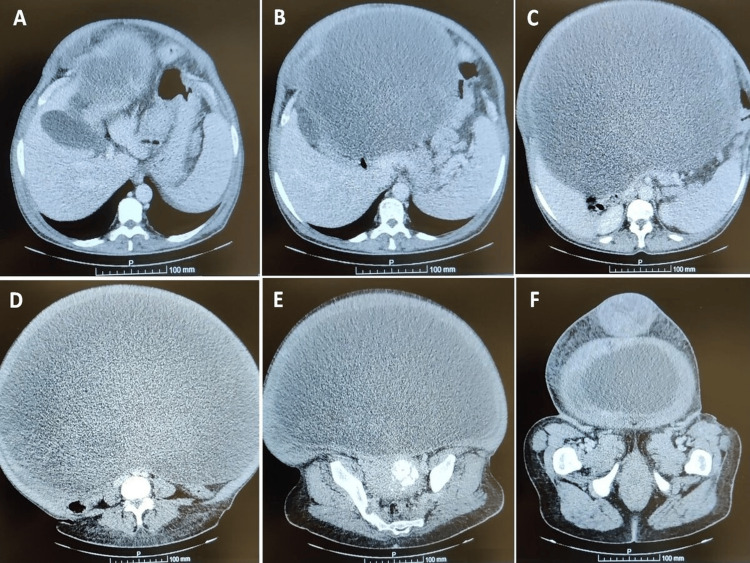
Multiple sections of the thoracic-abdominopelvic CT scan, oriented in a cephalocaudal direction.

In the emergency department, she underwent abdominal, pelvic, renal, and adrenal ultrasound examinations, revealing a suspicion of disseminated peritoneal disease of uncertain origin. Urinalysis showed no abnormalities. Laboratory tests revealed microcytic anemia. A chest X-ray demonstrated elevation of both diaphragmatic domes. She was admitted to the internal medicine service for additional evaluation, with the consideration of peritoneal carcinomatosis as a potential diagnostic hypothesis. Throughout the hospitalization, she remained hemodynamically stable. A thoracic-abdominopelvic CT scan was performed, revealing an extensive neoplasm, predominantly cystic, occupying the entire abdominal and pelvic cavity, with a largest diameter of approximately 43 cm (Figure 2). The neoplasm appeared well-defined and suggestive of a cystic ovarian neoplasm, and no ascites or lymphadenopathy was observed. The Risk of Ovarian Malignancy Algorithm score was calculated at 34.7% (cancer antigen 125: 155.6 U/mL, human epididymis protein 4: 37.3 U/mL). She was hospitalized for 12 days, receiving one unit of packed red blood cells due to anemia. There were no further complications during her stay, and her care was subsequently transferred to the Gynecology service due to suspicion of an ovarian tumor.

On 28/07/2021, she underwent exploratory laparotomy which revealed a massive right adnexal mass. Right adnexectomy was performed, and extemporaneous examination reported no signs of malignancy. Additionally, a total hysterectomy, left adnexectomy, and umbilical hernioplasty were performed. The massive abdominal mass measured 45 cm and weighed 35 kg and was regarded as the right adnexa. The procedure occurred without any complications. According to the pathological report, the mass corresponded to a leiomyoma with cystic degeneration and hemorrhage, suggestive of a parasitic leiomyoma.

During her 10-day hospitalization, she participated in a rehabilitation program, under the guidance of the Physical Medicine and Rehabilitation department, aimed at addressing her balance issue, likely stemming from her altered center of gravity.

**Figure 3:**
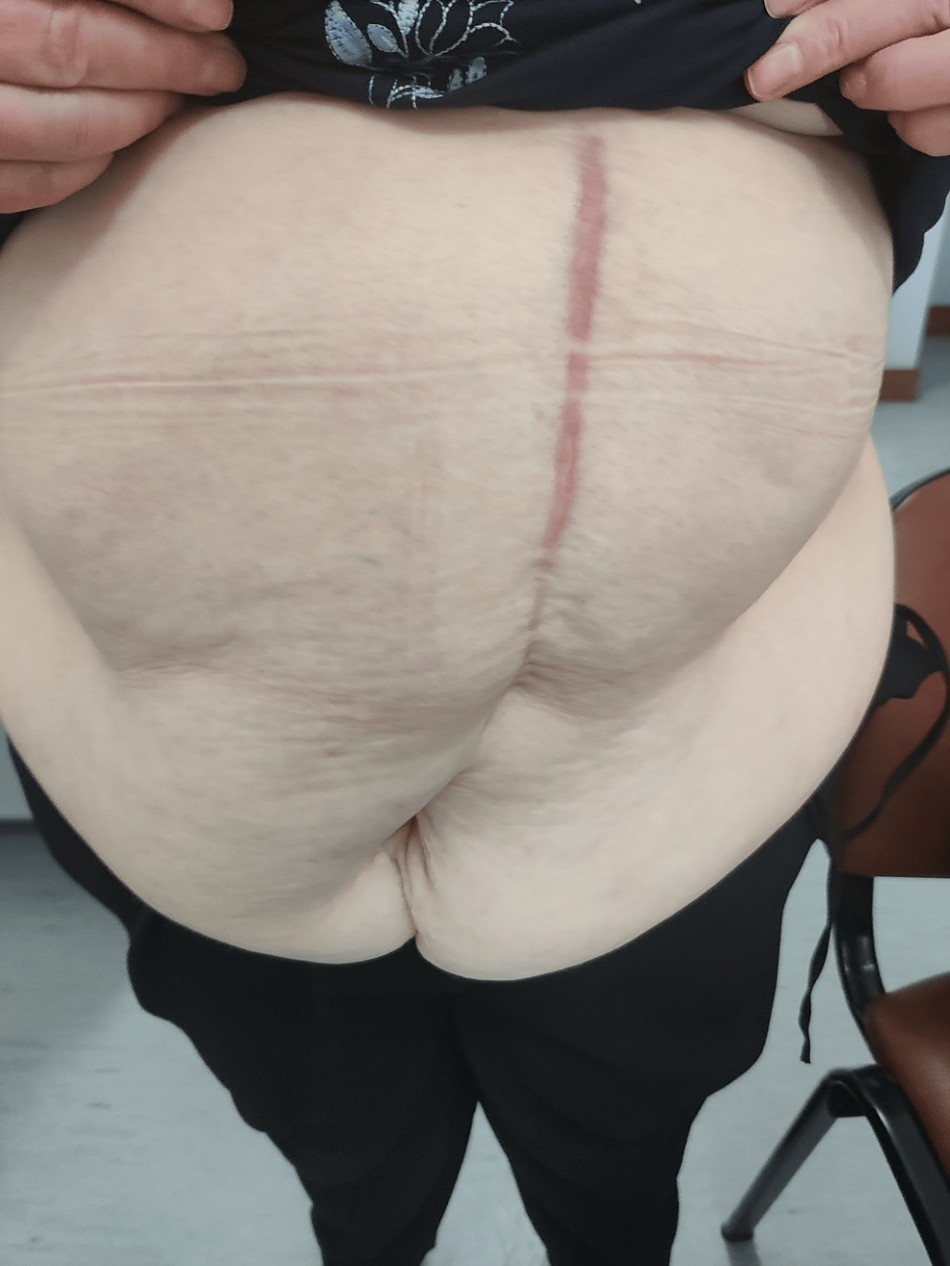
Photo taken five months post-surgery during a routine hypertension consultation with an abdominal circumference measuring 124 cm, weight of 87 kg (post-surgery weight gain of approximately 27 kg), and body mass index of 37.7 kg/m2.

Following this, the patient noticeably escalated her visits to the Family Physician and made the deliberate choice to entrust the management of her hypertension to the Family Physician (Figure 3), and the other members of her family also experienced an upsurge in the frequency of their visits to the primary healthcare facility.

## Discussion

Traditionally, parasitic leiomyomas are described as unusual variants of pedunculated leiomyomas that, for reasons unknown, exist outside the uterus in the abdominal cavity, surviving by obtaining a blood supply from neighboring structures. These leiomyomas are linked with non-specific clinical signs and symptoms and are typically discovered incidentally during surgery for another primary reason [4].

Leiomyomas affect several women at some point in their lives, underscoring their significant impact on women’s quality of life and emphasizing their importance for clinicians [5]. The natural regression of leiomyomas typically begins during menopause [6]. However, it is essential to note that this pathology may still persist in this age group [6]. There are rare cases, such as the one presented here, of postmenopausal parasitic leiomyoma. Although the reasons why some uterine fibroids regress while others do not during this stage of life remain unclear, hormonal factors are believed to be involved [6]. To date, differentiating between leiomyomas and leiomyosarcomas through imaging alone remains quite challenging and often nearly impossible [4,6].

The process of cervical cancer screening offers a unique opportunity to comprehensively evaluate patients, especially those who may not frequently engage with primary healthcare services. In this case, the patient’s decreased contact with healthcare services due to the pandemic and her non-frequent attendance at primary care appointments demonstrate the potential for screening programs to bridge gaps in healthcare utilization.

## Conclusions

Cervical cancer screening transcends its primary purpose of early cancer detection. It combines the principles of comprehensive, patient-centered care by affording the chance to evaluate patients who might otherwise slip through the healthcare system’s cracks. As primary healthcare providers, it is our responsibility to seize these opportunities and leverage them for the benefit of our patients’ holistic well-being.
